# The Effects of a Single Dose of Methylphenidate on Motor Performance in Young Healthy Participants Diagnosed with Attention-Deficit/Hyperactivity Disorder (ADHD)

**DOI:** 10.1192/j.eurpsy.2025.2093

**Published:** 2025-08-26

**Authors:** A. Mimouni Bloch, Y. Bloch, S. Tsuk

**Affiliations:** 1 Child Development Center, Loewenstein Hospital, Raanana; 2 Faculty of Medicine, Tel Aviv University, Tel Aviv; 3 Child and adolescent outpatient clinic, Shalvata Hospital, Hod Hasharon; 4 Exercise physiology, The Levinsky-Wingate Academic College, Netanya , Israel

## Abstract

**Introduction:**

Methylphenidate (MPH), a common stimulant medication for attention-deficit hyperactivity disorder (ADHD), has been shown to enhance both fine and gross motor skills in individuals with ADHD.

**Objectives:**

This study aims to assess the acute effects of a single dose of MPH on agility and balance motor tests, as well as physiological parameters such as heart rate (HR) and blood pressure (BP) at rest and after motor tests, in young healthy participants diagnosed with ADHD.

**Methods:**

A randomized crossover design was employed to evaluate agility and balance motor performance with and without MPH treatment. The study included 35 physical education students (mean age 25.3±3.6 years) diagnosed with ADHD, 65.7% of whom were male. Each participant used their prescribed MPH medication. Motor tests included a zigzag agility test, with and without leading a ball, and a balance test. Physiological measurements, including HR, BP, and core temperature, were recorded at rest and after the motor tests. Subjective sense of effort was evaluated using the RPE scale.

**Results:**

The agility test without a ball showed a small but significant improvement following MPH treatment (without MPH 7.4±0.8, with MPH 7.4±0.8, p= 0.25) . No significant differences were found in the balance test or the agility drill with a ball, regardless of MPH treatment. Systolic blood pressure (SBP) at rest was significantly higher after MPH administration (without MPH 126.7±10.8, with MPH 131.9±12.7, p=.023). There were no significant differences in diastolic blood pressure (DBP), HR, or core temperature at rest or after the motor tests.

**Conclusions:**

A single dose of MPH improved performance in the agility test without a ball but did not enhance balance or agility with a ball. These findings suggest that while MPH may enhance certain motor functions, its effects are selective and not universally beneficial across all motor tasks.

**Disclosure of Interest:**

None Declared

